# Investigation of a Possible Role for the Histidine Decarboxylase Gene in Tourette Syndrome in the Chinese Han Population: A Family-Based Study

**DOI:** 10.1371/journal.pone.0160265

**Published:** 2016-08-16

**Authors:** He Dong, Wenmiao Liu, Meixin Liu, Longqiang Xu, Qiang Li, Ru Zhang, Xin Zhang, Shiguo Liu

**Affiliations:** 1 Department of Anesthesiology, the Affiliated Hospital of Qingdao University, Qingdao, China; 2 Prenatal diagnosis center, the Affiliated Hospital of Qingdao University, Qingdao, China; 3 Department of Cardiac Ultrasound, the Affiliated Hospital of Qingdao University, Qingdao, China; 4 Department of Clinical Laboratory, the Affiliated Hospital of Qingdao University, Qingdao, China; 5 Department of Andrology, the Affiliated Hospital of Qingdao University, Qingdao, China; 6 Department of Respiratory Medicine, the Affiliated Hospital of Qingdao University, Qingdao, China; 7 Genetic Laboratory, the Affiliated Hospital of Qingdao University, Qingdao, China; Duke University, UNITED STATES

## Abstract

Tourette syndrome (TS) is a polygenic neuropsychiatric disease. Previous studies have indicated that dysregulation in the histaminergic system may play a crucial role in disease onset. In this study, we investigated the role of the histidine decarboxylase gene (*HDC*) in TS susceptibility in the Chinese Han population. After genotyping 241 TS nuclear families trios, we analyzed three tag *HDC* single nucleotide polymorphisms (rs854150, rs854151, and rs854157) in a family-based study using the transmission disequilibrium test (TDT) and haplotype relative risk (HRR). TDT showed no over-transmission in these SNPs across the *HDC* region (for rs854150: χ^2^ = 0.472, *P* = 0.537, OR = 1.097, 95%CI = 0.738–1.630; for rs854151: χ^2^ = 0.043, *P* = 0.889, OR = 1.145, 95%CI = 0.767–1.709; for rs854157:χ^2^ = 0.984, *P* = 0.367, OR = 1.020, 95%CI = 0.508–2.049). HRR also showed the same tendency (for rs854150: χ^2^ = 0.211, *P* = 0.646, OR = 1.088, 95%CI = 0.759–1.559; for rs854151: χ^2^ = 0.134, *P* = 0.714, OR = 0.935, 95%CI = 0.653–1.339; for rs854157:χ^2^ = 0.841, *P* = 0.359, OR = 1.206, 95%CI = 0.808–1.799). Additionally, the haplotype-based haplotype relative risk showed a negative association. Although these findings indicate an unlikely association between *HDC* and TS in the Chinese Han population, a potential role for *HDC* cannot be ruled out in TS etiology. Future research should investigate this more thoroughly using different populations and larger samples.

## Introduction

Tourette syndrome (TS) is a neuropsychiatric disease presenting as multiple motor and vocal tics that begin during childhood. Most individuals, upwards of 45%–60%, are also affected by a co-morbidity such as obsessive compulsive disorder (OCD), attention deficit hyperactivity disorder (ADHD), depression, or autism[1]. Although the disorders originating from TS sometimes lessen with age, the co-morbid symptoms may continue until adolescence and even adulthood [2,3]. Therefore, the occurrence of multiple conditions may present a challenge to the complicated pathogenesis of TS. The precise etiology of TS is poorly understood, although previous studies have suggested that it involves an interaction between genetics and the environment [4,5]. Studies of candidate genes have typically focused on neurotransmitters and neuromodulators, and dopaminergic neurotransmission, with dopamine receptors D1–D4 and dopamine β–hydroxylase often deemed causative loci of TS [6]. However, a recent study identified a rare mutation (p. W317X,c.951G>A) in the histidine decarboxylase gene (*HDC*) in a two-generation pedigree of a family with a high incidence of TS [7];this indicates a role for histaminergic neurotransmission in the onset and development of TS.

*HDC* is located on chromosome 15q21–q22. It is divided into 14 exons varying in size up to 24 kb, and encodes a 622-amino acid L-histidine decarboxylase, which is the rate-limiting enzyme in histamine biosynthesis[8]. The histaminergic (HA) neurons, projected broadly throughout the brain, are responsible for motor control and the regulation of sensory information, cognition, and attention [9]. Therefore, dysregulation in HA neurotransmission may contribute to certain neuropsychiatric conditions such as schizophrenia, ADHD, and narcolepsy[10]. Since the original mutation (W317X) in *HDC* was reported, the HA system has gradually come into focus as a potential pathogenic locus for TS. *hdc* knockout mice have shown traits relevant to features of TS, such as a deficit in prepulse inhibition and dysregulated dopaminergic innervations of the basal ganglia [11]. Subsequently, a significant association between *HDC* and TS was found in affected individuals from Canada, Germany, and Italy by detecting polymorphisms. However, no remarkable findings were observed in studies of individuals from Greece, Hungary, Poland, Albania, or Spain [12]. This shows that association analyses can draw inconsistent results that are limited by differences in race and sample size.

In the present study, we investigated the role of *HDC* in TS in a Chinese Han population using a family-based association study. Three tag *HDC* single nucleotide polymorphisms (rs854150, rs854151, and rs854157) were analyzed by the transmission disequilibrium test (TDT) and haplotype relative risk (HRR) in 241 TS nuclear families. In view of potential genetic heterogeneity, these analytical approaches may provide an authentic result without the influence of confounding factors.

## Materials and Methods

### Subjects

The study project was approved by the Human Ethics Committee of the Affiliated Hospital of Qingdao University, and informed written consent was given by all participants or their legal guardians. A total of 241 TS nuclear families trios were recruited from the Affiliated Hospital of Qingdao University and Linyi People’s Hospital, China, from October 2008to July 2015. All patients were independently diagnosed by two experienced psychiatrists according to the criteria of the Diagnostic and Statistical Manual of Mental Disorders, fourth edition, text revision (DSM-IV).

### Genotyping analysis

After collecting blood samples from all participants, genomic DNA was extracted using the Qiagen DNA extraction kit (Duesseldorf, Germany). Alleles of *HDC* SNPs (rs854150, rs854151, and rs854157) were detected by TaqMan allelic discrimination real-time PCR. Taqman probes and primers were designed by Applied Biosystems of Life Technologies. For rs854150, forward and reverse primers were 5′-GATCAAAAAATCTGGACGTATAAAG-3′ and 5′-CTGACAGGGCTCTTCTTTACCTTGA-3′, respectively; for rs854151, forward and reverse primers were 5′-GACAGAGAGCACTGTAAAATCTCCA-3′ and 5′- ACTCCTAGGAATGAACCCCGGGAAG-3′, respectively; and for rs854157, forward and reverse primers were 5′-TCTCCAAGCCTTCTCTTACTTACTT-3′ and 5′-TCACAGGACCCTATGGGGTAGGACC-3′, respectively. PCR was conducted in a 25 μl reaction mixture containing 1.25 μl 20 × SNP Genotyping Assay Mix, 12.5 μl 2 × PCR Master Mix, and 11.25 μl DNA in DNase-free water. Amplifications were carried out using a C1000TM thermal cycler system with the following conditions: 95°C for 3 min, followed by 45 cycles of 95°C for 15 s and 60°C for 1 min. All steps were carried out with relevant guidelines and regulations, and we could detect the fluorescent signal from VIC- or FAM-labeled probes in each cycle. Genotype discrimination was conducted using Bio-Rad CFX manager 3.0 software.

### Selection of SNPs and statistical analysis

We selected three tag SNPs (rs854150, rs854151, and rs854157) according to the HapMap CHB population. The linkage disequilibrium of these SNPs was 0.763, 0.287, and 0.282, respectively, indicating that they were independently inherited from each other. All data were analyzed by statistical software package SPSS21.0. Allelic and genotypic distributions of trios were tested using HRR and TDT. To increase the efficiency, we also assessed cases by haplotype-based haplotype relative risk (HHRR).

## Results

The study group consisted of 241 patients (190 males, 51 females; mean age, 9.29± 3.27 years). Genotyping results of all nuclear families trios were in accordance with Hardy–Weinberg equilibrium and Mendelian transmission, suggesting that the population was genetically balanced and could be used for association studies (for rs854150:χ^2^ = 0.199,*P* = 0.655; for rs854151:χ^2^ = 0.641, *P* = 0.423; and for rs854157: χ^2^ = 0.013, *P* = 0.909).

The genotypic distributions of rs854150 (CC, CG, and GG) were 103(42.7%), 107(44.4%), and 31(12.9%), respectively, in TS patients, and 212(44.0%), 212(44.0%), and 58(12.0%), respectively, in parents. For rs854151, AA, AG, and GG genotypes of patients were 112(46.5%), 101(41.9%), and 28(11.6%), respectively, and 223(46.3%), 204(42.3%), 55(11.4%), respectively, in parents. GG, AG, and AA genotypes of rs854157 were detected in 172(71.4%), 62(25.7%), and seven (2.9%) patients, respectively, and in 351(72.8%), 121(25.1%), and 10(2.1%) parents, respectively. TDT did not reveal a significant over-transmission of alleles for these polymorphisms (for rs854150: χ^2^ = 0.472, *P* = 0.537, OR = 1.097, 95%CI = 0.738–1.630; for rs854151: χ^2^ = 0043, *P* = 0.889, OR = 1.145, 95%CI = 0.767–1.709; and for rs854157: χ^2^ = 0.984, *P* = 0.367, OR = 1.020, 95%CI = 0.508–2.049). The HRR also indicated the same tendency (for rs854150:χ^2^ = 0.211, *P* = 0.646, OR = 1.088, 95%CI = 0.759–1.559; for rs854151: χ^2^ = 0.134, *P* = 0.714, OR = 0.935, 95%CI = 0.653–1.339; and for rs854157: χ^2^ = 0.841, *P* = 0.359, OR = 1.206, 95%CI = 0.808–1.799). Genotypic and allelic frequencies of the three polymorphisms are shown in Tables 1 and 2.

**Table 1:** TDT test results of three genetic loci in 241 trios.

Non-transmitted allele	rs854150			rs854151			rs854157		
		C	G		A	G		A	G
Transmitted allele	C	212	101	A	219	105	A	11	67
	G	111	58	G	102	56	G	56	348
TDT results									
Χ^2^	0.472			0.043			0.984		
*P*-value	0.537			0.889			0.367		
OR	1.097			1.145			1.020		
95%CI	0.738–1.630			0.767–1.709			0.508–2.049		

**Table 2:** HRR results of three genetic loci in 241 trios.

Group	rs854150 (G>C)		rs854151(A>G)		rs854157(G>A)	
	C(+)	C(-)	G(+)	G(-)	A(+)	A(-)
Transmitted allele	103	138	130	111	71	170
Non-transmitted allele	108	133	134	107	62	179
Results						
Χ^2^	0.211		0.134		0.841	
*P*-value	0.646		0.714		0.359	
OR	1.088		0.935		1.206	
95%CI	0.759–1.559		0.653–1.339		0.808–1.799	

To increase the efficiency of testing, we enlarged the cases by HHRR but observed similar results (for rs854150: χ^2^ = 0.462, *P* = 0.497, OR = 0.912, 95%CI = 0.698–1.190; for rs854151: χ^2^ = 0.042, *P* = 0.837, OR = 1.029, 95%CI = 0.786–1.345; and for rs854157:χ^2^ = 0.982, *P* = 0.322, OR = 1.196, 95%CI = 0.839–1.704). These results are listed in Table 3.

**Table 3:** HHRR results of three genetic loci in 241 trios.

Group	rs854150(G>C)		rs854151(A>G)		rs854157(G>A)	
	C	G	A	G	G	A
Transmitted allele	313	169	324	158	404	78
Non-transmitted allele	323	159	321	161	415	67
Results						
Χ^2^	0.462		0.042		0.982	
*P*-value	0.497		0.837		0.322	
OR	0.912		1.029		1.196	
95%CI	0.698–1.190		0.786–1.345		0.839–1.704	

## Discussion

TS is a complex neuropsychiatric illness resulting from an interaction between genetics, the environment, immunology, and hormonal factors[13]. Twin, family, and association studies have revealed important findings about the underlying genetic component of TS [14],but the specific cellular and molecular mechanism remains elusive. Candidate TS gene studies have typically focused on genes encoding parts of the aminergic modulatory systems, especially the dopaminergic system. Many other neurotransmitters have also been proposed as potential susceptibility genes of TS, such as those of the glutamatergic, cholinergic, GABAergic, and serotonergic systems [15]. However, Ercan-Sencicek et al. recently detected a rare mutation in a TS family in which the father and eight of his children were affected with the disease [7]. The mutation was a heterozygous G-to-A transition in exon 9 of *HDC*, which resulted in a premature termination codon (W317X). The same mutation was not detected in 3000 controls, indicating that it may be responsible for the autosomal dominant inheritance in the two-generation pedigree.

The signaling pathway of histamine is mediated by four G protein-coupled receptors (H1–H4) involved in the pathophysiology of TS that are mapped to the striatum and cortex [16]. In particular, the H3 receptor has been revealed as an important regulator of dopamine and serotonin, which have been studied more extensively as important loci for TS [17]. At the same time, the HA system was also found to be associated with other neurodegenerative diseases such as Parkinson’s disease and Alzheimer’s disease, and has been used as a therapeutic target to improve cognitive function [18].

*Hdc* knockout mice received widespread attention as an informative pathophysiological model of TS, in which *hdc* intron 5 to exon 9 was replaced by an external gene, leading to a decrease in histamine synthesis [19]. Although spontaneous tic-like movements were not detected, *hdc* knockout mice recapitulated the key features of TS when exposed to high-dose psychostimulants and acute stress [11]. They also showed a reduction in pre-pulse inhibition, which may be caused by increasing levels of dopamine [20]; this symptom was consistent with TS patients carrying the W317X mutation in *HDC*. Modelling this mutation in an animal greatly strengthened our view of its causative role in TS, and improved the understanding of the role of the HA system in the etiology of TS.

A recent genome-wide scan for *de novo* or transmitted rare copy number variations in TS has shown an enrichment of genes within histamine receptor signaling pathways [21]. Lei et al. identified three variants (IVS1 +52C>T, c.426C>A, and c.1743G>A) by screening all *HDC* exons in 100 Chinese Han patients with TS [22]. At the same time, a study carried out in 520 European nuclear families also indicated a strong allelic over-transmission ofrs854150 andrs1894236 in *HDC*. However, limited by the various races, these results were only observed in Canadian, German, and Italian samples rather than those from Greece, Hungary, Poland, Albania, or Spain. However, all subsequent studies have provided strong evidence that dysregulation in the HA system may contribute to the risk of TS.

In light of this evidence, we investigated the association of *HDC* and TS in a large sample of 241 trios originating from China. We analyzed three tag SNPs (rs854150, rs854151, and rs854157) across the *HDC* region using TDT and HRR. Our work was based on a family study to avoid the influence of confounding factors and to better reflect the efficacy of the experiment. Our results suggest that there is no significant association between *HDC* and TS in the Chinese Han population. However, the present study has a number of limitations, such as ethnic variations and the small sample size. Additionally, the three SNPs we studied are not sufficient to represent the whole gene. Moreover, the onset of TS may be influenced by hundreds of candidate genes with small individual effects [23]. Thus, future work should verify the role of *HDC* in TS in different races with larger sample sizes, and pay more attention to the effect of genetic clusters. Nevertheless, our work may motivate further studies to focus on the role of HA in TS, while an explicit understanding of the correlation between them may open up new prospects for therapeutic options for TS.
